# Initial investigations of a novel bioluminescence method for imaging dental demineralization

**DOI:** 10.1002/cre2.402

**Published:** 2021-01-28

**Authors:** Christopher Longbottom, Bruce Vernon, Emma Perfect, Anne‐Marie Haughey, Adam Christie, Nigel Pitts

**Affiliations:** ^1^ Dental Innovation and Impact King's College London London UK; ^2^ Calcivis Nine Edinburgh BioQuarter Edinburgh UK; ^3^ LUX Assure Ltd Heriot Watt Research Park Edinburgh UK; ^4^ Fraunhofer Centre for Applied Photonics The Technology and Innovation Centre Glasgow UK

**Keywords:** activity, calcium, caries, luminescence

## Abstract

**Objectives:**

In this in vitro study, a bioluminescent marker was investigated for its potential to illuminate the assessment of dental caries and dental erosion, which are significant clinical and public health problems, through its binding of those ions, notably Ca^2+^, known to be released during the process of demineralization.

**Materials and Methods:**

The light output from the selected bioluminescent marker was investigated in several experiments, including: (a)contact with a range of Ca^2+^ ion concentrations; (b) treatment of extracted teeth with solutions of differing pH, followed by application of the bioluminescence marker to assess Ca^2+^ ion release; and (c) application of the marker to freshly extracted teeth with natural and artificially created caries lesions on occlusal and smooth surfaces to image the Ca^2+^ ion distribution.

**Results:**

The results of: experiment (a) showed that the light output from the marker increases with increasing Ca^2+^ concentration and of experiment (b) showed increases in light being observed as increasingly acidic solutions were applied. The results of experiment (c) showed the bioluminescence images of the extracted teeth produced “demineralization maps” of the imaged surfaces.

**Conclusions:**

These results demonstrate the ability of a novel bioluminescence technology to image Ca^2+^ ions on tooth enamel surfaces which has potential in dental caries and dental erosion applications and provides the scientific basis for the ongoing development of that novel technology.

**ABBREVIATIONS**
CSP: calcium sensitive photoprotein
CSP‐a: calcium sensitive photoprotein aequorin
EFF: endoscopic filtered fluorescence
QLF: quantitative light fluorescence
S0: the ground state
S1: first excited singlet state
S2: second excited singlet state
T1: the lowest excited triplet state.
Ca(OAc)_2_
: calcium acetate
SEM: scanning electron microscope

## 1 INTRODUCTION

**FIGURE 1:**
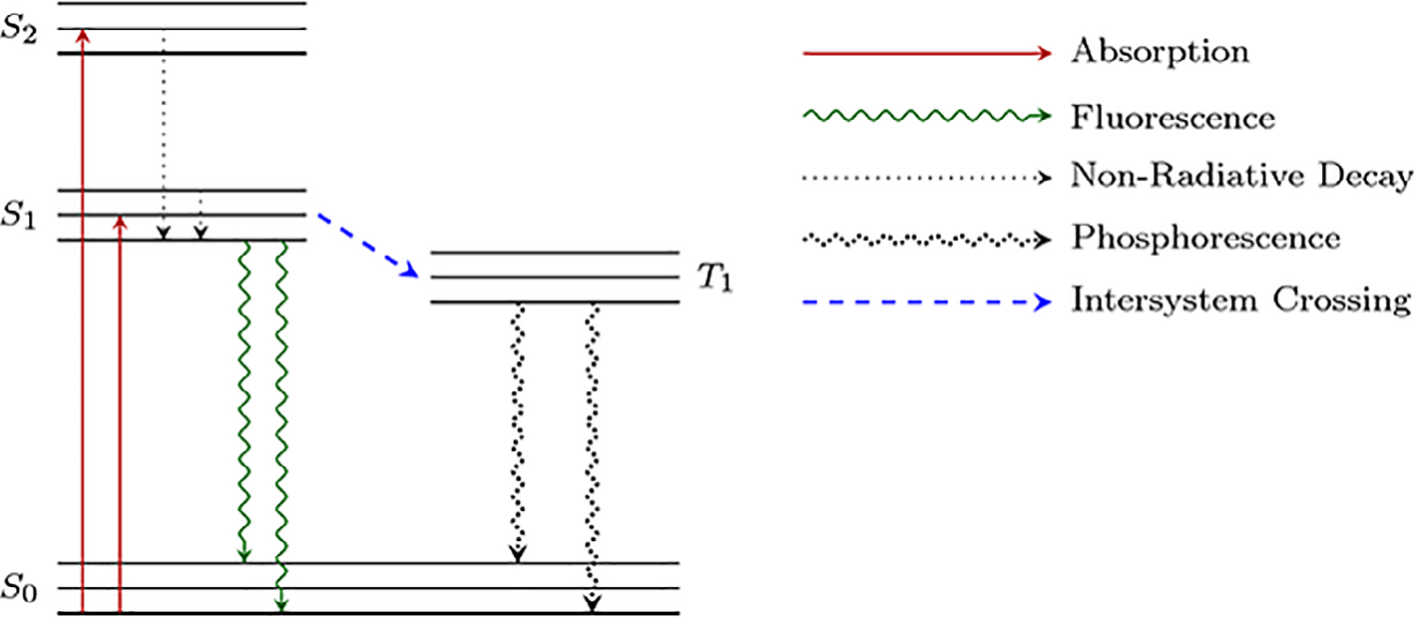
S0, S1 and S2 represent the ground state, first excited singlet state and second excited singlet state respectively and T1 represents the lowest excited triplet state. For fluorescence emission, electrons are excited from S0 to either S1 or S2. The electrons decay, non‐radiatively, to the ground level of S1 before decaying, radiatively to S0. For phosphorescence emission, which includes bioluminescence, electrons excited via chemical reaction transfer energy to T1 through intersystem crossing. The electrons then decay, radiatively, from the ground level of T1 to S0

There are a variety of medical techniques that utilize the properties of light for detection, diagnosis and treatment of disease(Ackroyd et al., 2001; Daniell & Hill, 1991; Harvey, 1993). In dentistry, there has been an increase in the use of light‐based technologies for detection of dental caries(Alfano & Yao, 1981; de Josselin de Jong et al., 1995; Jablonski‐Momeni et al., 2010; Jost et al., 2015; Lussi et al., 1999; Lussi et al., 2004; Neuhaus et al., 2009; Pitts & Longbottom, 1987; Schneiderman et al., 1997). Many of these use the principles of fluorescence to identify regions of the tooth that contain bacterial products such as porphyrins (DIAGNOdent) or location of demineralized dental tissue (endoscopic filtered fluorescence [EFF], quantitative light fluorescence [QLF]). Other studies from the 1970s and 1980s have used dyes, including fluorescent compounds, for caries detection (Alfano & Yao, 1981; Brooke et al., 1972; Daniell & Hill, 1991; de Josselin de Jong et al., 1995; Hardwick & Manley, 1952; Jablonski‐Momeni et al., 2010; Jost et al., 2015; Konikoff & Lyles, 1969; Lussi et al., 1999, 2004; Neuhaus et al., 2009; Pitts & Longbottom, 1987; Schneiderman et al., 1997; Sullivan, 1954; Van de Rijke, 1991a, 1991b). We present a light‐based approach that employs the properties of bioluminescence for the assessment of caries lesion *activity*, rather than detection. The general physical principles of bioluminescence are similar to those of fluorescence but specific aspects differentiate this technique from fluorescence approaches. Both fluorescent and bioluminescent light emission is a result of excitation of electrons from the ground state energy level to a higher vibrational energy level, see Figure 1 (Lakowicz, 2006). In both cases, light emission occurs when the excited electron returns to the ground state. A significant difference between the two techniques is the source of energy for the excitation of electrons. In the case of fluorescence, excitation is a result of absorption of a specific wavelength of light and an excitation source, such as a laser, is required. For bioluminescence, the energy required for excitation is produced by a chemical reaction between the bioluminescent agent and metal ions, e.g. calcium ions; therefore, a specific excitation light source is not required for the emission of a bioluminescent signal.

There are several key advantages in using bioluminescence rather than fluorescence as a tool to aid diagnosis in dentistry. As bioluminescence results from the direct reaction with calcium ions as they are lost from demineralizing enamel vis a vis sound enamel, it is producing images relating to the actual disease process at an ionic/molecular level rather than to historical scars from a previous episode of the disease process. Often, bioluminescence methods are more sensitive than fluorescence because the background light level for bioluminescence is generally lower than that of fluorescence, as there is no stray light from an excitation beam or auto‐fluorescence from samples captured by the detector. This increased sensitivity means that it is usually possible to make more precise measurements of small changes in light output which can be critical in identifying the early stages of disease development.

An issue for some previously used fluorescent and colorimetric dyes(Schroder, 1985; Rawls & Owen, 1978) has been their hazardous nature, preventing their intra‐oral use; toxicity testing has indicated that this is not expected to be a problem for the bioluminescent marker used here. With fluorescent dyes, the excess must often be removed after penetration into the lesion in order to measure fluorescence only from lesions(Van de Rijke, 1991a); this adds time and complexity to the procedure, making them difficult to use in vivo. Since the bioluminescent marker used here only generates a signal on binding Ca^2+^, the presence of excess marker is not a problem and does not need to be removed. Finally, as the bioluminescent light signal released from the calcium sensitive photoprotein (CSP; WO/2008/075081, n.d.) marker is directly related to the demineralization of the tooth, rather than indirectly through changes in tooth structure due to demineralization, the light signal is not significantly compromised by surface properties of the tooth, such as staining or the auto‐fluorescence of restorative materials. All current fluorescence‐based caries lesion detection systems are susceptible to the confounding effects of lesion staining which limit their sensitivity and specificity values, thereby considerably limit their application to monitoring of lesion behavior over time, that is, their applicability to lesion activity assessment.

Dental caries results in the net demineralization of tooth enamel in a complex process, which includes the sub‐surface regions, and leads to the release of ions, including Ca^2+^ (Larsen et al., 1997). The bioluminescence technique introduced here consists of applying a specific CSP—aequorin (hereafter referred to as “CSP‐a”)—to a tooth surface—a light signal is only emitted upon binding to solvated Ca^2+^ cations. If the tooth is undergoing net demineralization (by definition an active lesion) a light signal is expected to be observed; where no net demineralization is occurring, (by definition a sound tooth surface or an arrested lesion), minimal free Ca^2+^ will be present and little or no light is expected. Similarly, following consumption of acidic foodstuffs, such as soft drinks, Ca^2+^ is released from dental hard tissues into the surface micro‐environment, including in the enamel micro‐pores and this bioluminescent approach may also be useful to aid investigations of dental erosion (acid erosion).

This article presents the first data collected as part of feasibility studies into erosion and initial caries for this novel bioluminescence approach. Implications for caries lesion assessment, food‐stuff testing and dental product development are discussed.

## 2 EXPERIMENTAL

**FIGURE 2:**
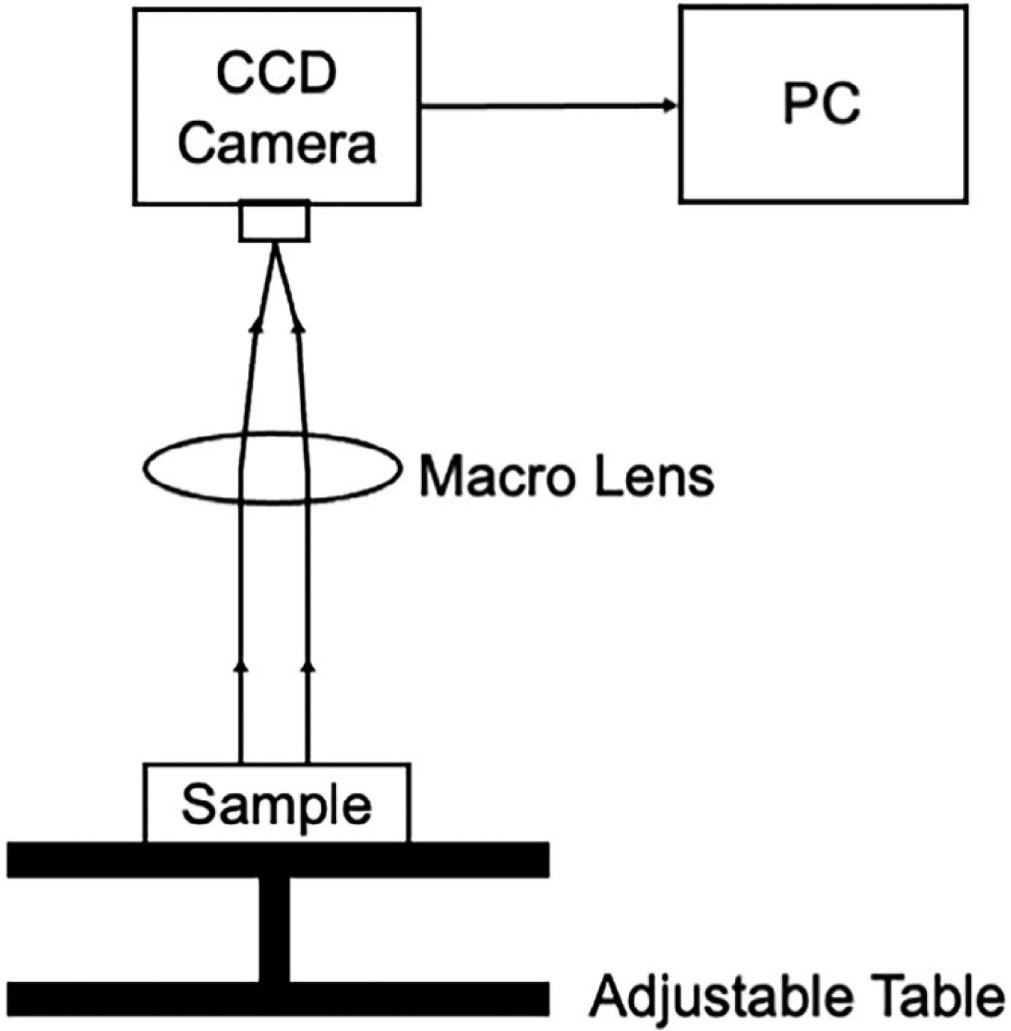
The adjustable table, sample, camera and lens are all housed within a dark box. The macro lens (1:25 90 mm Tamron) focal length is permanently set to 0.93 m and focus is achieved by altering the distance of the sample to the lens. The camera is connected to the PC, via USB, which is outside the dark box

Each sample, consisting of a whole tooth or hemi‐section, depending on the experiment—see below—was placed on a sample table in a dark box below a charged couple device camera and macro lens as shown diagrammatically in Figure 2.

### 2.1 Determining calcium concentration versus total light output

It was important initially to determine the relationship between calcium ion concentration and the total light output.

Ten different concentrations (0.01, 0.02, 0.05, 0.1, 0.2, 0.5, 1, 2, 5 and 10 mM) of Ca(OAc)_2_ (calcium acetate) were prepared in de‐ionized water. A volume of 10 μl of each solution was transferred to a small Petri dish and left to evaporate on the bench (approximately 30 min); the position of each dried spot was noted. The Petri dish was placed in a dark box below a camera (HX916, Starlight Express) as shown in Figure 2. The bioluminescent marker used in the following experiments, CSP‐a, (100 μl), was injected onto the dried spot and images from five consecutive camera exposures (60 s each) were acquired. The total light output was calculated by summing the mean greyscale values from each of the five exposures using Image J (Rasband & ImageJ, 1997‐2016). Five replicates of each concentration were analyzed; the results were averaged and the mean standard deviation calculated.

### 2.2 Assessment of demineralization potential of drinks and solutions of different pH with CSP‐a

Thirty teeth were selected at random from a batch of teeth identified as sound by an experienced clinician (C Longbottom). Teeth throughout this work were supplied by the University of Dundee and obtained in accordance with national and local ethics committee guidelines. A line of nail varnish was painted over the enamel‐dentinal junction to delimit the crown and the root. Solutions of varying acidity were selected and the pH measured (pH meter 8424, Hanna Instruments): 1% citric acid (pH 2.3), lemon juice (pH 2.5), diet cola (pH 3.1), apple juice (pH 3.2), 0.01% citric acid (pH 3.3), Ribena Really Light (pH 3.8), orange juice (pH 4.0), 0.0001% citric acid (pH 4.9), deionized water (pH 5.5), semi‐skimmed milk (pH 6.7), phosphate buffered saline (pH 7.2) and sodium hydroxide (pH 13.5). Prior to the application of a test solution, each tooth was imaged (60 s exposure) in a dark box after the application of CSP‐a (250 μl). CSP‐a was rinsed from the tooth with deionized water and the tooth immersed in a test solution (4 ml) for 120 s at room temperature. The tooth was removed from the test solution and rinsed briefly with deionized water. The tooth was then re‐imaged in the dark box (60 s exposure) after the addition of CSP‐a (250 μl). Images generated were analyzed for mean light output (grayscale value). Experiments were performed in triplicate, using fresh teeth and mean standard deviations calculated.

### 2.3 Artificial caries lesion creation

Carboxymethylcellulose gel (3% wt/vol Akucell AF 1985 in deionized water) was prepared at either pH 4.7 or pH 6.4 using lactic acid to modify pH. Ten teeth were selected at random from a pool of teeth supplied by the University of Dundee. Enamel windows (approximately 4 × 4 mm^2^) were created on a smooth surface of extracted premolar teeth by placing a paper label on the tooth and painting around the label with colored nail varnish. The label was removed after the nail varnish had dried, thereby creating a “window” of enamel. Gel (either pH 4.7 or 6.4) was applied (250 μl) to the window and the tooth incubated in a hydrated environment at 37°C for 10 days. Five teeth were used for each of the different pH levels. After incubation, the gel was gently removed from the tooth surface with tissue paper and the surface was rinsed with deionized water. Each tooth was placed in a dark box and imaged under lit conditions and then in darkness after addition of 200 μL CSP‐a (60 s exposure time). Five replicates were used and for graphical representation results were averaged and mean standard deviations shown.

### 2.4 Caries activity maps

One hundred nineteen primary teeth, scheduled to be extracted from young children at the University of Dundee Dental School as part of individual treatment plans, were used in the study in accordance with national and local ethics committee guidelines and having first obtained the consent of the children's guardians. After extraction, the teeth were rinsed with deionized water, brushed gently occlusally with a toothbrush, and lightly flossed approximally by a clinician. A pencil mark was made by the clinician, an experienced pediatric dentist/cardiologist, on the root surface directly below where there was a visible approximal enamel caries lesion. The teeth were hemisected (Microslice 2 Precision Slicing Machine, Malvern Instruments) along the bucco‐lingual axis (for approximal surfaces) and along the enamel dentine junction (for occlusal surfaces) under wet conditions to prevent the tooth from drying out. Each sample was mounted (Bostik Superglue) on a carbon scanning electron microscope (SEM) stub (Agar Scientific) and imaged (DC500, Leica) in color. Each mounted tooth was then transferred to a dark box and imaged under lit conditions and then in darkness (HX916, Starlight Express) immediately after the addition of CSP‐a (250 μl).

### 2.5 Analysis of images

**FIGURE 3:**
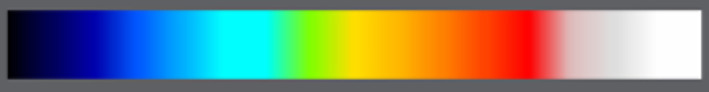
Color scale applied to images captured in darkness after the addition of CSP‐a (calcium sensitive photoprotein aequorin). The black end of the scale represents a greyscale value of 0 and the white end of the scale represents the highest greyscale value of 150

Luminescent images captured after the addition of CSP‐a were analyzed with Image J software using the following method. Images were false colored using the “Royal” color scale, shown in Figure 3, and the maximum greyscale value was decreased from 255 to 150. The purpose of false coloring and decreasing the greyscale value is to make luminescent light signal easier to visualize.

## 3 RESULTS

### 3.1 Calcium concentration versus Total light output

**FIGURE 4:**
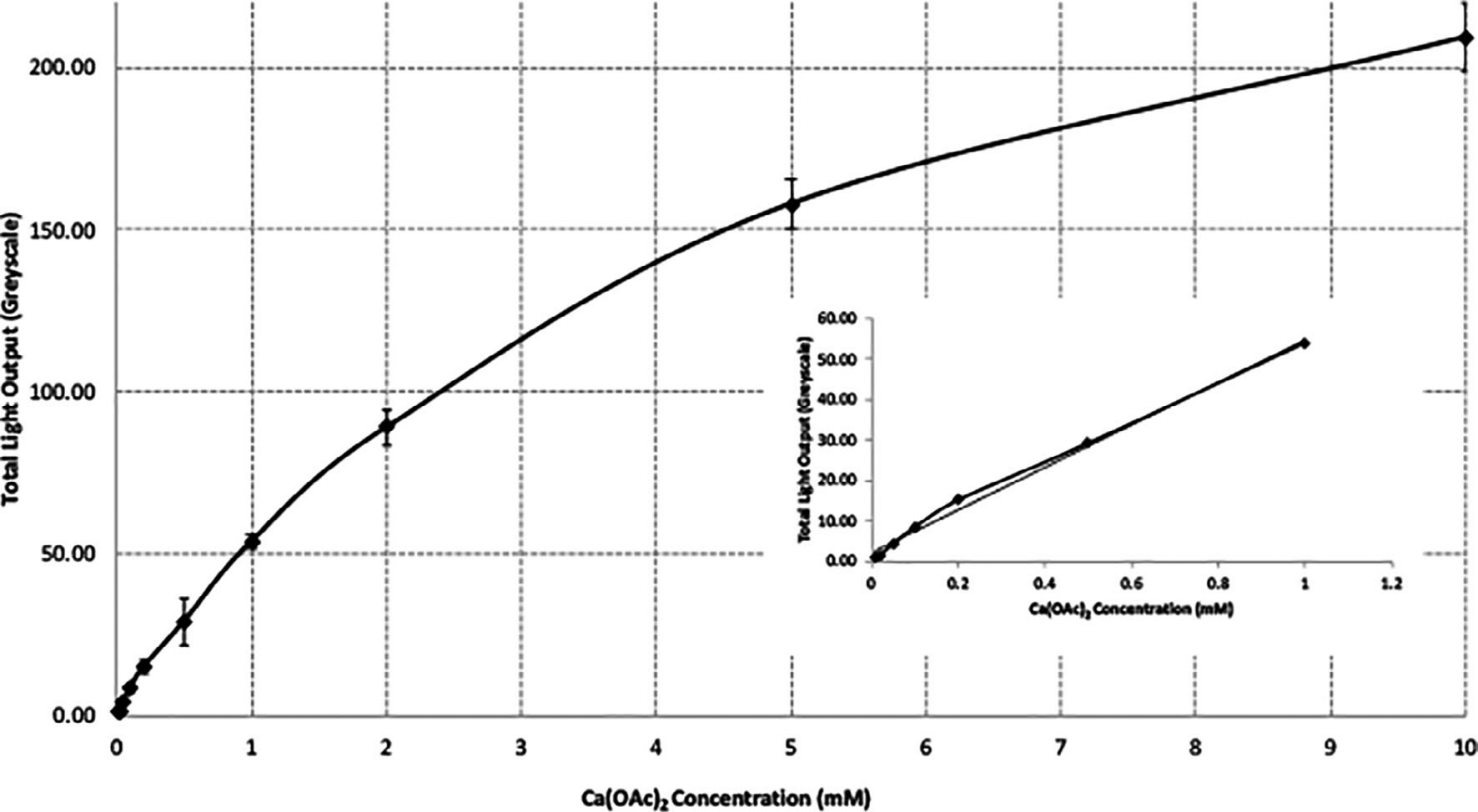
The relationship between concentration of calcium and light output as determined using different concentrations of dried Ca(OAc)2 (calcium acetate) spots

**FIGURE 5:**
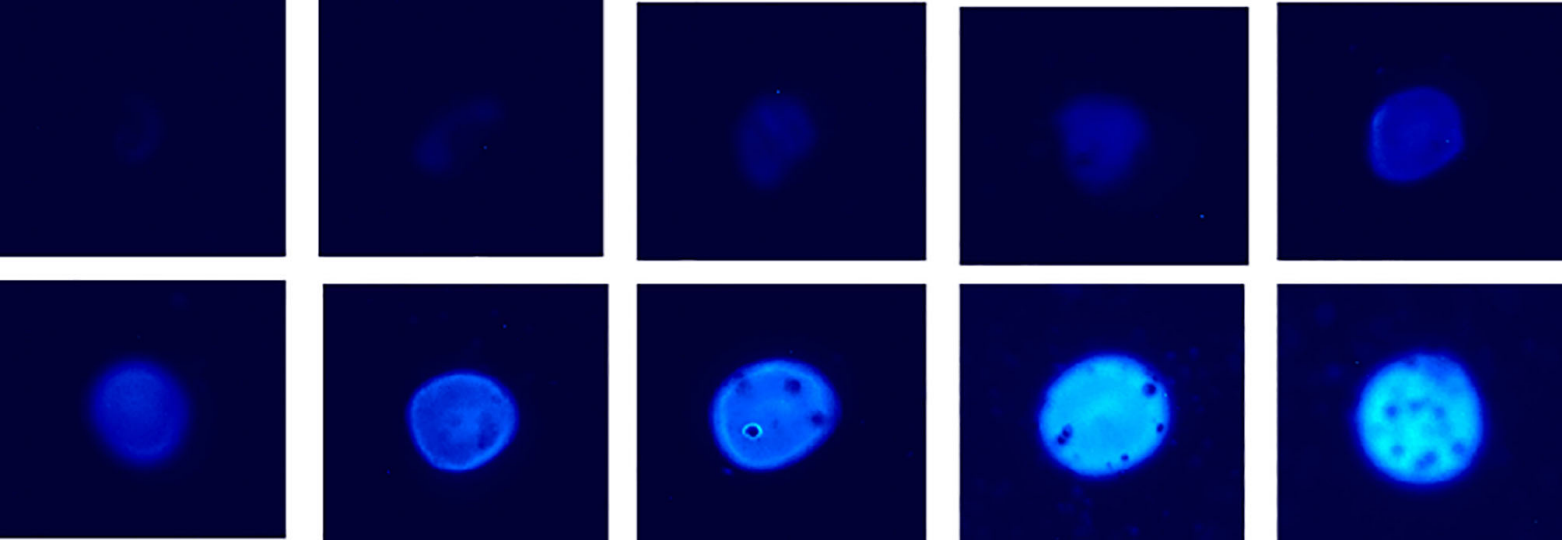
Light captured from CSP‐a added to increasing concentrations of Ca(OAc)2 spots (0.01, 0.02, 0.05, 0.1, 0.2, 0.5, 1, 2, 5 and 10 mM from top left to bottom right). Images have been false colored and show the summed total light of five consecutive 60 s exposures

The increase in CSP‐a light output with increasing Ca^2+^ concentration is approximately linear up to a concentration of 2 mM and plateaus at concentrations approaching 10 mM when dried Ca(OAc)_2_ was used, see Figure 4. Images of the light output from the various Ca^2+^ concentrations are also shown, see Figure 5.

### 3.2 Acid and drinks effect on the CSP‐a signal

**FIGURE 6:**
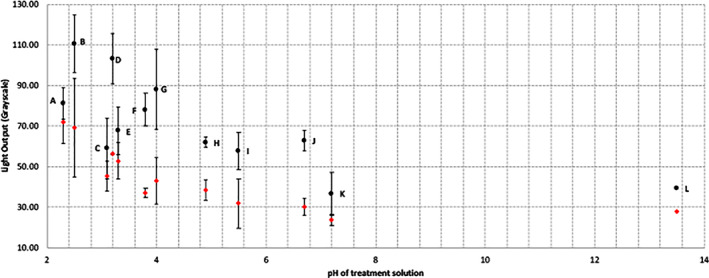
The relationship between solutions of varying pH and the light output from both the crown and root portions of the tooth after addition of CSPa. The solutions used from point a to point L are 1% citric acid (pH 2.3, a), lemon juice (pH 2.5, b), diet cola (pH 3.1, c), apple juice (pH 3.2, d), 0.01% citric acid (pH 3.3, e), Ribena really light (pH 3.8, f), orange juice (pH 4.0, g), 0.0001% citric acid (pH 4.9, h), deionized water (pH 5.5, i), semi‐skimmed milk (pH 6.7, j), phosphate buffered saline (pH 7.2, k) and sodium hydroxide (pH 13.5, l)

**FIGURE 7:**
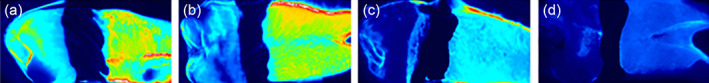
Teeth after treatment with 4 ml acidic solution (lemon juice, apple juice, orange juice and phosphate buffered saline from left to right) for 120 s and after addition of CSP‐a (250 μl). Image exposure time was 60 s

Figure 6 clearly shows a relationship between the pH of the solution used to treat a tooth and the light output following application of CSP‐a. The lower the solution pH the greater the light signal recorded—this occurred for both the crown and the root of the tooth, with the correlation significant at the 0.01 and 0.05 levels, respectively, using Spearman's rank correlation coefficient, but with a leveling off of light output when using solutions of pH 5.5 and above. Although light output from roots appeared to be greater than from the crowns this was not shown to be significant in all cases (one sided paired *t*‐test). Figure 7 shows images demonstrating the light output from four teeth after treatment with lemon, apple and orange juice or phosphate buffered saline and following application of CSP‐a.

### 3.3 Artificial caries creation and impact on CSP‐a signal

**FIGURE 8:**
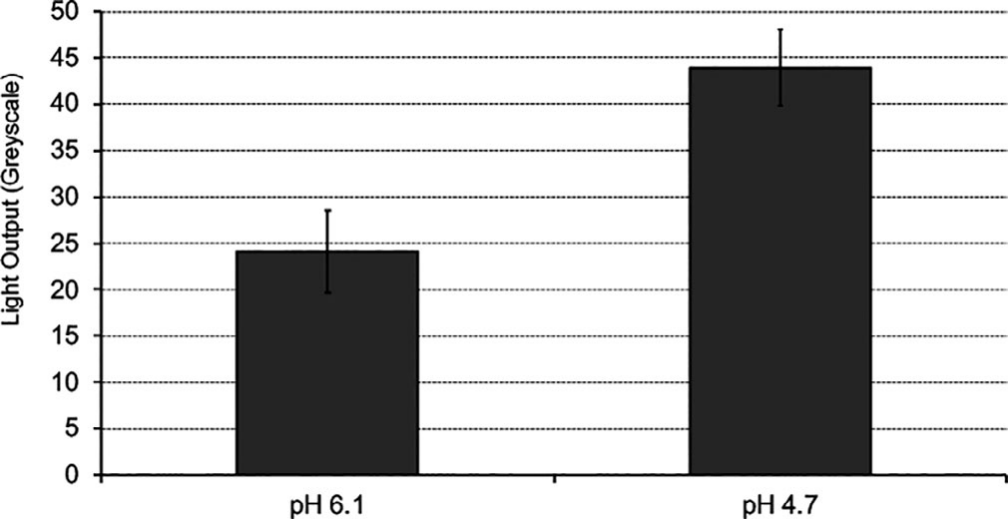
Average light output from five teeth demineralized for 10 days with acidic gel (pH 6.4 or 4.7) captured (60 s exposure) after the addition of CSP‐a (200 μl). Errors shown are the mean standard deviations

**FIGURE 9:**
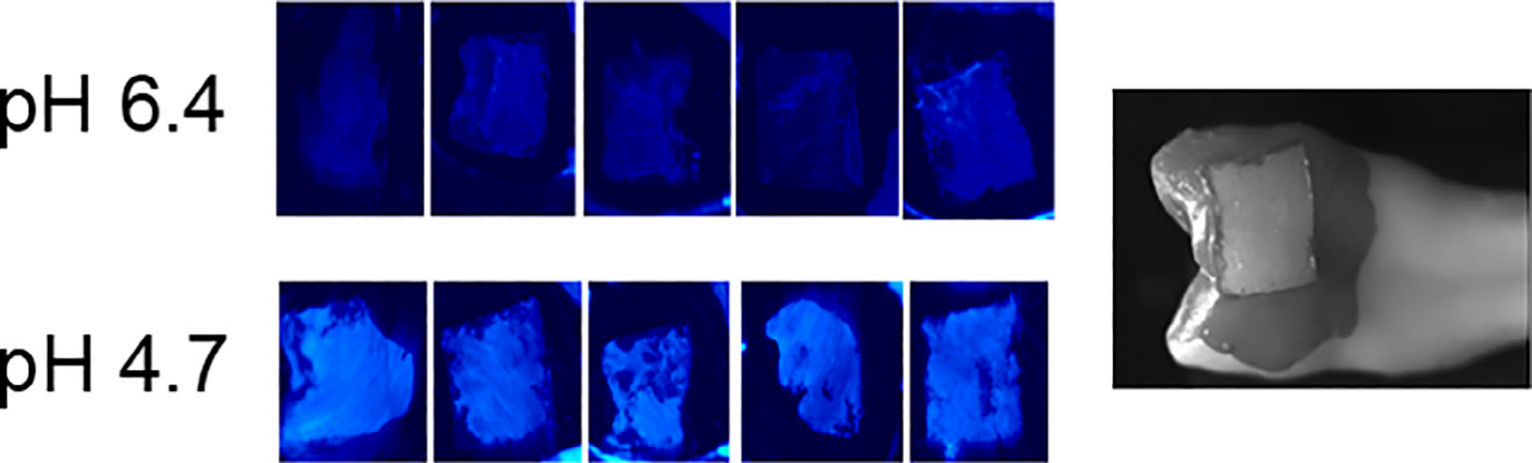
Luminescent images of the 10 demineralized teeth and a light image showing an example of the area that was demineralized on the tooth surface

Figures 8 and 9 clearly show that the teeth incubated with the gel of pH 4.7 resulted in a greater light output following the application of CSP‐a than those incubated with gel of pH 6.4.

### 3.4 Caries activity maps

**FIGURE 10:**
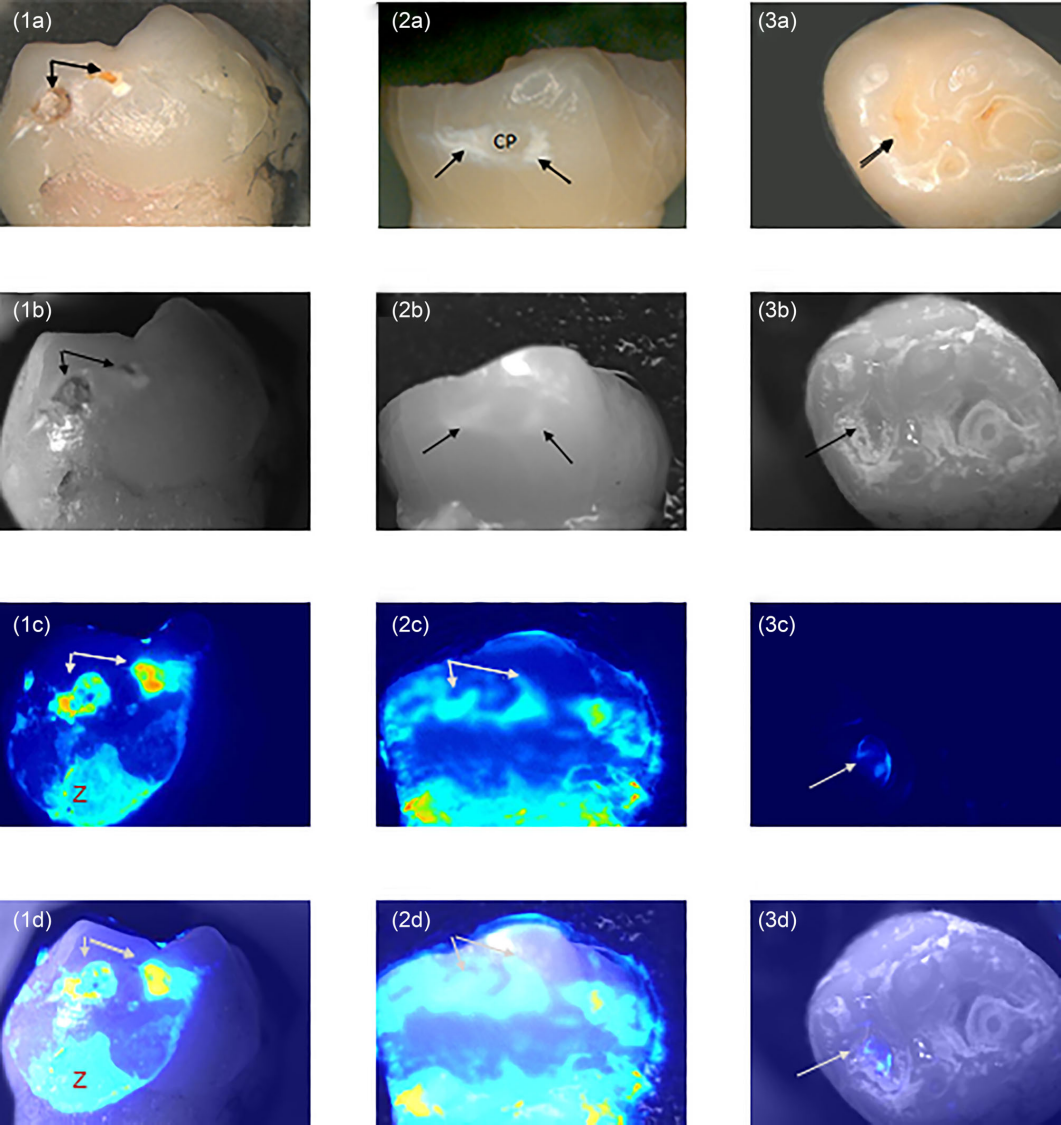
Color light images row “a,” black and white light images row “b,” luminescent images row “c” and merged “b” and “c” images row “d.” Luminescence images show regions of Ca2+ cations on the tooth surface and the merged images show the position of the luminescence on the tooth surface. In 1c and d “Z” indicates an area of tissue that has remained on the tooth after extraction and is producing a bright light signal. In 2a–d “CP” represents the contact point—the area that would have been in contact with the adjacent tooth when in vivo

From the more than 100 teeth imaged, three freshly extracted teeth are shown in Figure 10 as typical examples. Row “a” shows a color image of the tooth, row “b” shows a black and white image taken in the light, row “c” shows the luminescent image taken after the application of CSP‐a and row “d” shows “b” and “c” merged to demonstrate the position of the luminescence pattern in relation to the surface of the tooth. Images 1a–d and 2a–d show an approximal surface (which in the mouth contacts an adjacent tooth) and 3a–d show an occlusal (biting) surface. On the surface of tooth 1 (1a–d) two small cavities are visible where the outer enamel surface has lost its original surface integrity (arrows). The luminescent image of tooth 1 (1c) shows a brighter luminescent signal from the cavity regions and Figure 1d illustrates that the position of the two areas of brighter luminescence correspond to the position of the lesions. There is also a region of luminescence toward the enamel‐dentine junction (Z) which is the location of remnant soft tissue (visible as a faint pink area at the bottom of the photo in the Row A color image) that remained attached following extraction. Tooth 2 (2a–d) exhibits considerable tooth tissue loss from the occlusal surface of the tooth. 2a,b show a white spot lesion on the approximal tooth surface (arrows), the circular area within the white spot lesion corresponding to the contact area with the adjacent tooth before the imaged tooth was extracted. The luminescent image, 2c, shows a pattern of luminescence in the same shape as the lesion and image 2d confirms the coincident position of the luminescence pattern with the white spot lesion. Tooth 3 (3a–d) shows a very specific region of luminescence from within the fissure. This region was identified as active caries by a clinician after extraction, prior to luminescence imaging.

## 4 DISCUSSION

During the demineralization of enamel, as occurs during both the caries and erosion processes, ions, notably Ca^2+^, will be released. We have investigated whether a calcium‐sensitive bioluminescent marker, CSP‐a, could provide a useful tool with which to investigate caries and erosion. To be useful, the marker must generate a signal that varies depending on the concentration of ions present. Results presented here demonstrate that greater levels of ionic Ca^2+^ do generate a greater light response from CSP‐a. Significantly, the initial part of the curve is linear, suggesting that CSP‐a may be used relatively simply for quantification of ionic Ca^2+^, which could be important in the development of products such as a laboratory assay for the testing of foodstuffs and dental products. Furthermore, CSP‐a responds to very low levels of ionic Ca^2+^, which is likely to be crucial for investigation of the very early stages of caries.

Given these encouraging results we investigated applying CSP‐a to whole teeth that had been acid‐treated in order to investigate tooth erosion and the risk profile of various drinks. The luminescence signal was observed to increase as teeth were treated with solutions of decreasing pH, a result that is in agreement with previous work where pH was found to have a major influence on demineralization (Grenby et al., 1989; Shaw & Smith, 1999). The pH at which saliva, and more importantly plaque fluid ‐ are exactly saturated in phosphate with respect to enamel, is termed the critical pH, below which hydroxy‐apatite rods in enamel start losing minerals through the inter‐rod spaces, resulting in a reduction of the rod size, with a corresponding enlargement of inter‐crystalline spaces and an increase in enamel porosity. The critical pH of enamel is typically pH 5.2–5.5 but for dentin it is closer to pH 6.

The approach used to investigate erosion was simple to perform and is expected to translate easily into a laboratory assay with which to screen foodstuffs to assess their risk of generating erosion or in the development of novel dental products with which to protect enamel from erosion. With this approach the tooth surface itself can be assessed after treatment, as compared to using only the acid solution employed‐ this means that a method for screening opaque or colored solutions, which normally cause significant absorption and light scatter in light‐based assays, is feasible. Given that many foodstuffs fall within this category, this should offer a significant advantage over other assays. Further work is now required to investigate the assay more thoroughly, particularly in relation to the titratable acidity of the investigated substance, the pellicle and saliva, as well as to compare a CSP‐a ‐based assay with more traditional methods employed in dental erosion studies, such as atomic absorption spectroscopy and surface profilometry.

Regarding tooth decay, the artificial caries model employed in this study highlighted the potential of CSP‐a for use in lesion assessment, since greater luminescence was observed following use of acid gel compared than near‐neutral gel. These data ruled out the possibility that CSP‐a generates a signal from the Ca^2+^ that forms part of the hydroxyapatite crystal lattice itself, since in that case all tooth areas would display similar luminescence levels, regardless of gel acidity. Rather, light is apparently generated from the binding of CSP‐a with ionic Ca^2+^ generated during demineralization. Given these promising results, we applied CSP‐a to freshly extracted teeth where caries lesions were clearly visible clinically when examining the tooth out of the mouth and away from any close anatomical relationships with neighboring teeth. Distinct patterns of luminescence were observed, the location of which matched that of the visible lesions. In particular, Figure 10: 2a–d shows a pattern typical of many of the approximal surfaces assessed; at the contact area there will be an absence of plaque/biofilm and so active demineralization is highly unlikely. However, below (apically to) this contact area plaque/biofilm tends to build up, leading to possible demineralization and white‐spot lesion development. Therefore, there appears to be merit in further investigating this approach for the assessment of caries lesions and it may be of particular use for areas where assessment is problematic, including approximal surfaces, pits and fissures, as well as enamel adjacent to restorations. As with the erosion studies, further work is required to investigate potential confounders which are found in vivo, such as saliva and plaque/biofilm.

In conclusion, this novel photonics‐based method shows great promise for a variety of dental applications. Since carrying out the studies reported herein, our investigations of the technology have continued, and a key step has been incorporation of a CSP into a clinical product. Prior to in vivo use the toxicological properties of the chosen CSP was established. It is important to realize that direct comparative work with established caries lesion detection methodologies is not appropriate, since the CSP technology is designed to help assess lesion activity not lesion presence per se. The validation of lesion activity status presents significant methodological challenges but these can be approached using laboratory and clinical protocols designed to assess a number of factors, including lesion surface morphology and longitudinal monitoring of the staging of lesions. Last but not least, the optical array for in‐mouth signal collection will be different to the experimental setup described herein and presents challenges of miniaturization. As investigations have continued since these original experiments reported here, a different but related photo‐protein which is more sensitive and specific to calcium ions than CSP‐a has been investigated, tested for toxicological safety and a clinical device has been developed and tested in vivo. These more recent results will be reported separately.

## AUTHOR CONTRIBUTION

CL, EP, AM Haughey contributed to the conception of the study and the study design. All authors contributed to the interpretation of the results and to drafting the manuscript, including final approval.

## CONFLICT OF INTEREST

Dr. Longbottom reports personal fees from Calcivis Ltd., during the conduct of the study; personal fees from Calcivis, Ltd., outside the submitted work; In addition, Dr. Longbottom has a patent No. WO2008075081A2 issued. Dr. Pitts reports personal fees from Calcivis, Ltd., outside the submitted work; Dr. Vernon reports and Calcivis employee. Dr. Christie reports and Calcivis employee. Dr. Perfect reports grants from UK government TSB grant, during the conduct of the study; In addition, Dr. Perfect has a patent WO2008075081A2 issued. Dr. Haughey has nothing to disclose.

## Data Availability

The data that support the findings of this study are available from the corresponding author upon reasonable request.
